# Sex and gender differences in Lewy body dementia: a narrative review

**DOI:** 10.1016/j.neuros.2025.100009

**Published:** 2025-09

**Authors:** Ece Bayram

**Affiliations:** Movement Disorders Center, Department of Neurology, University of Colorado Anschutz, 12469 E 19th Ave., Aurora, CO, 80045, USA

**Keywords:** Sex, Gender, Lewy, Parkinson, Cognition, Female, Women

## Abstract

Lewy body dementia (LBD), including Parkinson’s disease dementia and dementia with Lewy bodies, is one of the most prevalent and burdensome type of dementias. Clinical diagnostic accuracy during life remains limited and there are currently only symptomatic therapy options without disease modification. However, recent advances in biomarkers and clinical trials are promising. Literature so far showed sex and gender differences in older adults without cognitive changes, people with all-cause dementia, Alzheimer’s disease, and Parkinson’s disease. While the number of studies in LBD are lower, understanding sex and gender differences and the underlying reasons can improve both diagnosis and treatment for LBD. Accordingly, the aim of this narrative review is to provide a summary of the literature for sex and gender differences in LBD. Majority of the studies for LBD investigating sex/gender differences so far focused on sex, with sex and gender terms being misused at times. Experiences of people in non-binary categories for sex or gender have yet to be investigated. While more research is needed, findings so far outline sex differences in prevalence, risk factors, biomarkers, symptoms, progression, treatment, daily life, and pathology for LBD. Sex-specific risk factors have also been reported, emphasizing the value of sex-stratified analyses and investigating female/male-specific factors such as sex hormones, menopause, and sex chromosomes. Lack of adequate research representation for females and women, as well as people from non-binary categories, is an important limitation that should be addressed to obtain more applicable findings in LBD.

## 1. Introduction

Lewy body dementia (LBD) is the second most common neurode-generative dementia, including Parkinson’s disease dementia (PDD) and dementia with Lewy bodies (DLB). PDD and DLB both require autopsy-confirmation for gold standard diagnosis and are clinically differentiated by the interval between the onset of dementia and parkinsonism [1]. There are currently no disease modifying therapies available and diagnostic accuracy during life remains limited for LBD [1]. Thus, LBD prevalence is likely higher than currently estimated, due to clinical underdiagnosis and Parkinson’s disease (PD) being the fastest growing neurological disease globally [2]. Raising awareness in addition to advances in biomarkers [3] and recently published diagnostic criteria for prodromal DLB [4] will hopefully boost diagnostic and therapeutic efforts in the near future.

Many studies use “sex” and “gender” terms interchangeably and pool the sexes and genders without considering potential differences. This makes it challenging to interpret the findings for applicability across different sex and gender groups [5]. Sex and gender are interrelated, but they refer to different concepts [6]. Sex primarily refers to biological factors including genetics, physiological and anatomical sex traits with female and male categories. A rare group of people can also have a variety of chromosomal compositions beyond XX/XY. Gender is a social construct related to norms, roles, and behaviors that vary over time and between societies. Gender identity may or may not correspond with the designated sex at birth.

Currently, experiences of females and women remain understudied in LBD. Thus, in this narrative review, I aimed to provide an overview of research for sex and gender differences to trigger discussions on the gaps, needs, and future directions for LBD. Sex (female/male) and gender terms (women/men) are reported as used in the cited work, unless misuse was determined based on the intended meaning within the scope of work or operational definitions. I also sought studies in LBD including non-binary categories for sex and gender.

## 2. Epidemiology

Global all-age prevalence of PD is 1.51 per 1000, with lower rates for females compared to males (1.49 vs 1.54 per 1000) [7]. This lower prevalence for females is consistent after the age of 50 on a global scale. Older age is one of the most reported risk factors for LBD and up to 80 % can progress to PDD after 20 years of PD [8]. Higher prevalence of PD for males coupled with higher risk for PDD with older age and longer disease duration, can result with higher PDD prevalence for males than females. Accordingly, PDD is typically more common for males than females [9].

The median time to dementia from PD diagnosis can differ by 6 years between females and males, showing faster progression for cognitive decline in males [9], further contributing to higher prevalence rates for males. On the other hand, sex differences for PD and potentially PDD prevalence can vary across regions. In the Western Pacific region and in countries with very high human development index and high sociodemographic index, PD prevalence was higher for females than males across all age groups [7]. Interestingly, a study with a German cohort showed while PDD is more frequent for males, this sex difference becomes insignificant once accounting for sex-specific survival patterns [10]. In a US cohort, females and males with PD had similar self-reported dementia risk, although the female advantage was significant once adjusting for the sex differences for age, education, and perceived discrimination while receiving healthcare [11]. Thus, social variables and gender disparities can impact the biological sex difference in prevalence rates.

Findings on sex differences for DLB prevalence is not as consistent as PDD. Overall, DLB accounts for ~5 % and PDD accounts for ~3.5 % of all dementia cases [8,12]. Some report higher prevalence for males, while some report the opposite [12,13]. Age and geographic location may play a role in these differences; prevalence seems to be higher for males than females for <75 years of age and in US cohorts [14,15]. Whether the study was done in a clinical or population cohort also plays a role, as clinical DLB cohorts include more males and sex ratio is more balanced in population studies [14].

## 3. Risk factors

Risk factors for PDD and DLB include various modifiable and non-modifiable risk factors with sex differences from genetics to childhood social status. Studies on all-cause dementia report that a considerable amount of dementia cases are likely preventable. These modifiable risk factors include low education, hypertension, obesity, diabetes, alcohol, smoking, depression, hearing loss, sensory loss, sleep disturbance, traumatic brain injury, social isolation, diet, vitamin D deficiency, and physical inactivity [16,17]. These factors differ by sex and gender throughout life with different effects on dementia risk. In the COSMIC Consortium, including over 45,000 people from 21 countries across Africa, Asia, Europe, North America, South America, and Oceania, women had lower education, higher likelihood of depression history; men had higher likelihood of smoking/alcohol history, and were more engaged in high physical activity [18]. While dementia incidence rates were similar across countries based on income levels for men; rates were the highest among women from low and middle-income countries. Obesity was associated with dementia risk only for men; alcohol use and lower years of education increased dementia risk more for men than women. These findings are based on the cohorts with all-cause dementia outcome without classification for different dementia subtypes [18].

For DLB, older age, smell loss, rapid eye movement sleep behavior disorder (RBD), autonomic dysfunction, hallucination, and depression were associated with higher risk [1]. For PDD, more consistently reported risk factors include older age, lower years of education, more severe motor impairment, postural instability gait disorder subtype, hallucinations, orthostatic hypotension, cerebrovascular disease, diabetes mellitus, obesity, cardiac disease, history of alcohol consumption, and smoking [19–21]. Physical activity is reported as a protective factor [20]. In a German cohort of people with PD, cerebrovascular disease presence increased the risk of PDD for both sexes but was more prevalent for males than females at PD diagnosis [10]. Age increased the incidence of PDD, particularly in females. Detrusor overactivity was associated with higher dementia risk for only females with PD in a Brazilian cohort [22]. In a US cohort of males with benign prostatic hyperplasia, *α*–1 adrenergic receptor antagonists that bind to and activate an adenosine triphosphate (ATP)-producing enzyme in glycolysis (terazosin, doxazosin, alfuzosin) were associated with lower DLB risk than medications that do not increase ATP (tamsulosin or 5*α*-reductase inhibitor) [23].

Disparities experienced more by women with PD can also conceal the female sex advantage for PDD risk [11]. In a US cohort, compared to males identifying as cisgender and heterosexual, females identifying as cisgender and heterosexual, and as sexual and gender minority had similar risk; males identifying as sexual and gender minority had higher risk for PDD. This higher risk for males identifying as sexual and gender minority disappeared; and female advantage became significant once accounting for age, education, and perceived discrimination while receiving healthcare. In another US cohort, we identified that different social determinants were associated with LBD risk for females and males [24]. Lower childhood subjective social status for females; being ethnoracially minoritized and lower years of education for males were significant risk factors.

The most recent work from the International LBD Genomics Consortium, consisting of participants with European ancestry, reported *GBA, BIN1, TMEM175, SNCA-AS1*, and *APOE* as risk factors [25]. Interestingly, these genes were not significant in a smaller study including a Japanese cohort [26]. This study identified another gene, *CDH23* as a risk factor [26], emphasizing the differences in genetic risk factors across populations. While *GBA* mutations were more prevalent in males than females with DLB in cohorts including participants of European ancestry [27,28], several studies noted similar prevalence by sex [29]. In the Pacific Udall Cohort, longitudinal analyses showed that males with *GBA* mutations had a higher PDD risk than females [30].

Sex-stratification might help identify significant loci with sex-specific effects. Risk variants may be concealed in the combined cohorts, due to significance in only one sex or opposite effects for the sexes resulting in non-significance. Accordingly, we conducted the X chromosome wide association study with the International LBD Genomics Consortium and found a significant variant (*MAP3K15*, rs141773145) only for females, which was not significant in the model combining females and males [31]. The effect levels of risk loci can also differ by sex; in AD, sex-specific polygenic hazard scores perform better than hazard scores based on the combined female and male cohorts for age at onset, clinical progression, and pathology [32]. In the National Alzheimer’s Coordinating Center (NACC) data from the US, *APOEe4* association with PDD risk did not differ by sex [33]. However, sample sizes for genetic studies in PDD and DLB are substantially limited compared to AD. With harmonization and international consortia efforts in PD and LBD [3,25,34,35], future studies may have more power to perform sex-stratified analyses.

## 4. Clinical profile

A typical LBD phenotype includes non-amnestic dementia, well-formed visual hallucinations early in the dementia process, cognitive fluctuations, RBD, and parkinsonism (Table 1) [36,37]. Memory deficits, mood and behavior changes, other sleep disturbances, autonomic dysfunction, and smell loss can also occur and support the diagnosis. Studies so far support sex differences for clinical profile prior to, at, and after dementia onset as well as the progression rate.

Compared to males, females seem to have a delayed clinical diagnosis for DLB [38]. Females are less likely than males to present with a typical LBD phenotype at dementia onset, contributing to this delayed diagnosis. A study with a Chinese cohort assessing initial symptoms retrospectively for people with probable DLB reported memory loss as the most frequent initial symptom for both females and males [39]. Psychiatric symptoms, including visual and auditory hallucinations were more common; RBD was less common for females than males. Although not significantly different than males, delusion, depression, and autonomic symptoms were more common, apathy and parkinsonism were less common for females as well. A number of LBD studies from Eastern and Western populations have shown that females experience RBD and parkinsonism less than males [14]. For females, visual hallucinations are more common than males in clinically defined cohorts; and less common than males in pathologically defined cohorts [14]. Majority of studies reported no difference for cognitive fluctuation prevalence. In a multicenter Chinese cohort and a Dutch cohort, prevalence of all core features (visual hallucination, cognitive fluctuation, RBD, and parkinsonism) was similar for females and males with DLB or PDD [40,41].

In prodromal DLB, bradykinesia, rigidity, RBD, and cognitive fluctuations were more prevalent for males than females in the NACC [42]. Similarly, compared to females, parkinsonism was more prevalent for males with prodromal DLB, and RBD was more prevalent for males with prodromal PDD in a Chinese cohort [40]. However, in this cohort, cognitive fluctuations were more common in females than males with prodromal DLB, despite the opposite finding in the NACC [40,42].

Cognitive profile is often used to differentiate between different dementia types such as LBD and AD, even in the prodromal stages [43]. For PD, sex differences for cognition is similar to the reports in healthy populations with females performing better on verbal learning and memory tests, and worse on visuospatial function tests [44,45]. Interestingly, females and males with prodromal DLB were reported to have similar cognitive profiles [42], with potentially lower scores on verbal fluency for females prior to and after dementia onset [41,42].

Neuropsychiatric symptoms are more common for females than males with PDD and DLB prior to and at dementia onset [10,40]. A meta-analysis on neuropsychiatric features across alpha synucleinopathies (combining PD, PDD, and DLB) showed that females have a higher prevalence of pyschosis, anxiety, depression, and fatigue; and males have a higher prevalence of apathy, impulse control disorders, RBD, hypersomnolence, and suicide [46]. In a Japanese cohort, males with DLB were more likely to experience hyposmia, syncope at diagnosis, while females were more likely to experience auditory hallucinations [47]. Prevalence of autonomic disturbance, including orthostatic hypotension and constipation, which commonly occur in LBD, did not differ by sex [47].

### 4.1. Progression

Compared to females with PD and normal cognition, males may progress faster to MCI and PDD [44]. After dementia onset, several studies reported similar rate of decline on global cognitive screening measures (i.e., Mini Mental State Examination) by sex for DLB, despite some supporting faster cognitive decline for females and some for males [14]. In a German cohort, males had higher mortality rates than females with PDD across all age groups [10]. In the NACC, there were no sex differences for mortality rates for people with LBD [48]. In a Turkish and a US cohort, females had higher mortality rates and survived for a shorter period of time than males with DLB after dementia diagnosis and admission to a nursing home [49,50].

### 4.2. Daily impact

The impact of symptoms on daily life can differ across individuals with LBD. Male sex has been associated with more impairment in activities of daily living despite similar prevalence and severity of behavioral and motor symptoms in DLB [51]. Compared to people with normal cognition and similar age in the US, income and subjective social status can be lower for people with LBD, with a larger decline for females than males [24]. In the US, misdiagnosis of dementia substantially impacts care costs, care access, and well-being [52]. This can have important implications particularly for females with LBD given higher delayed and misdiagnosis rates.

While the experience of caregiving likely differs based on both the person living with LBD and the caregiver’s characteristics, not much is known in terms of sex and gender differences. For both men and women with LBD, majority of the caregivers are women and include mostly spouses, a lower number of daughters, and daughters-in-law [14,53]. For dementia, female caregivers experience higher burden than males [54]. However, a study in a Norwegian cohort reported similar levels of caregiving burden for female and male caregivers of people with DLB [55].

## 5. Biomarkers

A range of biomarkers including structural and functional neuroimaging, neurophysiology, biofluid and skin biomarkers can support clinical diagnosis and detect the underlying pathology during life for LBD.

### 5.1. Neuroimaging

A cross-sectional study including a multi-site European and a US cohort, showed that males with DLB had more widespread cortical atrophy including frontal cortex, and females with DLB had thinner olfactory cortices on magnetic resonance imaging (MRI) [56]. These sex differences may be age specific; significant differences were noted for females and males younger than 75 with similar disease duration, while males and females with DLB over the age 75 had similar regional gray matter volume and cortical thickness [56]. In a US cohort, females with DLB had more white matter hyperintensities than males with DLB on MRI [57].

Dopamine transporter single photon emission computed tomography (DaTscan) and metaiodobenzylguanidine (MIBG) myocardial scintigraphy help assess neuronal loss in Lewy body diseases. Dopamine transporter and MIBG uptake is reduced in PDD and DLB, and both imaging markers have high diagnostic accuracy for the typical LBD phenotype [58]. A study from Switzerland with ^123^I-FP-CIT SPECT (DaTscan) reported that compared to males with DLB, females with DLB had lower binding in the extrastriatal projections of the nigrostriatal and mesolimbic dopaminergic systems [59]. Connectivity of nigrostriatal and mesolimbic systems was impaired for both females and males; however, patterns differed by sex. Females had long-distance disconnections between subcortical and cortical regions, and males had local alterations.

Both females and males with DLB can have typical DLB-like hypometabolism on (18)F-fluorodeoxyglucose positron emission tomography (FDG-PET), that involves the occipital, temporoparietal, and dorsolateral prefrontal cortex [60]. However, sex differences for FDG-PET patterns also occur for DLB. Compared to males, females have more severe brain connectivity alterations, and less severe occipito-parietal hypometabolism. Metabolic connectivity findings showed noradrenergic and dopaminergic network changes in both females and males, with cholinergic network changes only in males.

Slower pattern on electroencephalogram (EEG) and magnetoencephalogram can help support the LBD diagnosis. Sex differences for resting-state EEG (rsEEG) were shown in a multi-site European cohort [61]. For PDD, males had more abnormalities of widespread delta and alpha rsEEG activities. For DLB, males had more abnormalities of central-parietal delta and posterior alpha rsEEG activities. For both PDD and DLB, sex hormones impacted the neurophysiological mechanisms and the generation of rsEEG rhythms.

### 5.2. Biofluids

Alpha-synuclein, amyloid, and tau pathology, autophagy lysosomal pathway, ubiquitin proteasome system, mitochondrial dysfunction, neuroinflammation and oxidative stress have been noted in LBD pathophysiology [62]. Accordingly, biofluid markers for these mechanisms can serve as biomarkers for LBD. A recent meta-analysis reported that for people with DLB, cerebrospinal fluid (CSF) tau, neurofilament light chain (NfL), CHI3L1 and glial fibrillary acidic protein (GFAP) levels were higher compared to people with normal cognition [63]. CSF alpha-synuclein levels were lower for people with DLB compared to people with normal cognition or other dementias, without significant differences for alpha-synuclein levels in plasma or serum. For PDD, CSF tau and NfL levels were higher, and alpha-synuclein levels were lower compared to people with normal cognition. These biomarkers may look different for females and males, as LBD pathophysiology and underlying pathologies may differ by sex. In healthy aging, oxidative and inflammatory stress mechanisms alongside inflammatory markers differ by sex [64]. For DLB, females had lower CSF alpha-synuclein and amyloid*β*42 levels than males in the Amsterdam Dementia Cohort [41]. Sex-stratified comparisons of people with DLB to controls in Malmö Alzheimer Study, showed lower CSF alpha-synuclein and orexin levels particularly for females, supporting the utility for sex-specific biomarkers [65].

Alpha-synuclein real time quaking induced conversion (RT-QuIC) in CSF, skin, and olfactory mucosa are promising biomarkers in PD and DLB, without reports on sex-specific utility [66]. Furthermore, AD co-pathology does not seem to substantially impact the sensitivity of alpha-synuclein RT-QuIC, except for olfactory mucosa. Utilizing this technique can improve the clinical diagnosis particularly for females with underlying Lewy body pathology, as they have a higher risk of AD co-pathology and clinical misdiagnosis with AD.

## 6. Pathology

Lewy body pathology is required for the pathological diagnosis of LBD. AD co-pathology and substantia nigra neuron loss also frequently occur in LBD and are associated with the clinical profile (Table 2) [67]. The prevalence of these pathologies can differ by sex. In the NACC, pure neocortical Lewy body pathology was more frequent for males than females across different age groups [68]. Substantia nigra neuron loss was more severe for males than females, until the neocortical Lewy body stage [69]. In another US based cohort of community-dwelling older adults, after adjusting for age at death, education, race, and *APOEe4*, females were more likely to have AD and cerebrovascular disease pathologies than males, and males were more likely to have pure Lewy body pathology than females [70].

Whether associations between these pathologies differ by sex, if or how these pathologies occur sequentially based on sex are not clear. But these findings for prevalence are highly important since LBD phenotype is more likely to occur with neocortical Lewy bodies, more severe substantia nigra neuron loss and lower levels of AD co-pathology [37]. Consequently, one can assume females are less likely than males to present with a typical LBD clinical profile. In fact, the likelihood is even lower due to clinicopathological associations also differing by sex.

In the NACC, compared to males, females with pure Lewy body pathology were less likely to experience dementia and a typical LBD phenotype including cognitive fluctuations, visual hallucinations, RBD, and parkinsonism [71]. Once female and male cohorts were matched 1:1 for age, education, and Braak neurofibrillary tangle stage, females continued to be less likely to have a history of clinical LBD diagnosis. Including the people with AD co-pathology in the cohort showed that AD co-pathology was associated with lower LBD phenotype particularly for males, worse dementia severity and AD phenotype particularly for females [72]. More severe substantia nigra neuron loss correlated with parkinsonism and LBD phenotype more for males than females in models adjusted for age, Lewy body and AD pathology staging [69].

Since traditional staging approaches can fall short in capturing the extent of burden, we also assessed whether there are sex differences in clinical associations of regional Lewy body and AD co-pathology burden. Using a US based cohort of people with a high likelihood of LBD based on traditional pathology staging, females continued to be less likely to have a typical LBD phenotype despite more Lewy bodies than males [73]. Higher middle frontal, cingulate, entorhinal Lewy body counts were associated with higher LBD phenotype likelihood particularly for males.

These studies underscore the clinical underdiagnosis of LBD for females, and the need to better understand if females can withstand higher levels of Lewy body pathology before developing symptoms. Incorporating both Lewy body and AD biomarkers even if people have a typical AD phenotype can help identify females with Lewy body pathology with or without AD co-pathology more accurately.

## 7. Treatment

Recently, we have seen promising results from clinical trials in LBD [74]. However, for now, there is no cure or commercially available disease modifying treatments for LBD. A multidisciplinary approach utilizing pharmacological, non-pharmacological, and psychosocial strategies is recommended [75] (Table 3). Additionally, comorbidities that can contribute to cognitive decline and at times be treatable, medications that can trigger and worsen the cognitive and behavioral problems, modifications and support to improve the daily life, and specific needs of the people with LBD and their care partners should be considered. Prevalence and impact of comorbidities can differ by sex; in a Japanese cohort, females with dementia had lower vitamin B1 and B12 levels than females without dementia and B1 levels were associated with higher dementia risk only for females. Males with and without dementia had similar vitamin levels [76].

Typical (haloperidol) and some other antipsychotics (olanzapine, risperidone) should be avoided in LBD due to serious side effects [1]. Women with dementia are prescribed antipsychotic and other psychotropic drugs for longer periods than men [77]. The higher likelihood of psychiatric onset in prodromal stages and higher misdiagnosis rates for females than males with LBD, can put them at risk for prescription of these drugs [78]. Even in cohorts without dementia, antipsychotic treatment response differs by sex; females experience more side effects with antipsychotics and mood stabilizers than males [14,79].

The earlier occurrence of behavioral symptoms in LBD leads to increased need for care and institutionalization compared to AD [80]. Females tend to outlive males; and in a US-based cohort for LBD, females were more likely than males to live alone and not have a partner [24]. Thus, females may have to rely more on paid caregivers or institutions increasing the cost of care. In contrast, Medicare expenditures during the last five years of life were similar for females and males with LBD and similar dementia severity [81]. Nonetheless, care expands beyond what is covered by insurance; coverage, access and utilization can also differ for women and men. Exercise offers a home-based therapy option for people with LBD to maintain without significant adverse effects. Interestingly, exercise effect on cognitive decline was more pronounced in females than males in a US-based cohort of people with mild cognitive impairment [82]. In a UK-based cohort, cognition stimulation therapy improved cognition and quality of life more for women than men [83].

As effects of therapeutic approaches including both pharmacological and non-pharmacological options differ, lower representation of females in clinical trials puts them at a higher risk of mistreatment due to unknown benefits and side effects. Study designs should also consider the specific needs of women for inclusion in research. Many clinical trials requiring a co-participant can contribute to the lower rates of research participation for women with LBD, given higher likelihood of living alone without a partner [24]. Outcome measures relying on care partner report may differ based on both the affected individual and care partner sex and gender.

## 8. Best practices for future research

The need to include a more diverse group of participants in LBD research is clear from the existing studies. For equity in neurology research, Lizarraga and colleagues recently synthesized existing guidelines and statements to provide seven strategies including (1) learning history, (2) learning about upstream forces, (3) diversifying and liberating, (4) changing narratives and adopting best communication responses, (5) studying social drivers of health and lived experiences, (6) leveraging health technologies, (7) building, sustaining and leading culturally humble teams [84]. While they used ethnicity and race as main examples with a general neurology focus, these strategies also apply to sex and gender research in LBD.

Study teams should be aware of the history and barriers for LBD research participation in their targeted community [85]. While building relationships and trust with community members takes time and an ongoing effort, it is a much-needed investment for the success of research [86]. For recruitment, diversifying and tailoring recruitment materials and advertising approaches based on the community, building diverse research teams, partnering with people from the community, healthcare workers, and research advocates are effective [87]. Lack of awareness about research opportunities can limit participation, despite interest for people with LBD [85]. Thus, community education and outreach activities to inform the community about research can help boost recruitment and retention.

Even if people from minoritized groups are made aware of research opportunities, they can experience barriers based on study design [88]. Considering different research methods that are more accessible and inclusive can enhance participation and retention. For instance, requiring caregiver participation can limit the number of women with LBD participating in research. Compared to men, women with LBD may be more likely to have paid caregivers or their children as caregivers who are unable to show up for research visits during work hours [53,89]. It is important for the study team to be trained for implicit bias and to pay attention to using inclusive language. Study team members may make assumptions and fill out sex and gender identity questions without asking the participants [90]. In contrary to the presumption of older adults not willing to disclose their sexual orientation and gender identity status, our recent LBD survey study including sex, gender identity, and sexual orientation questions were filled out by all participants despite being provided with “Prefer not to disclose” response option [24]. The online data collection with deidentified data sharing in the Fox Insight [91] has allowed us to conduct studies on sexual and gender minoritized groups with PD [11,88,92]. Study teams should be mindful of wording used for the questions and responses, provide rationale for asking these questions with response options beyond binary, refer to participants with the preferred name and pronouns, and inform the participants about data privacy measures to protect their identity as disclosure of this type of sensitive information can put people at risk in certain communities [90].

For publishing, authors can refer to Sex and Gender Equity in Research (SAGER) guidelines [93]. Journals providing definitions for the terms and guidelines for transparent reporting of sex and gender can help with the accurate and consistent use of terminology in research from different cohorts [5]. Misuse of the terms leads to misunderstanding or misrepresentation of findings. Reporting sex and gender differences without considering the interaction between biological and social factors, or potential underlying causes for the differences can deepen the inaccurate stereotypes and disparities [94]. After publishing the study findings, sharing the output with the community can increase community’s trust in the study team, engagement in future research, and implementation of the findings. Advocacy for research and policies to address health disparities can be strengthened by identifying the underlying causes of sex and gender differences, and with the support of community members.

## 9. Conclusion

**Fig. 1.**
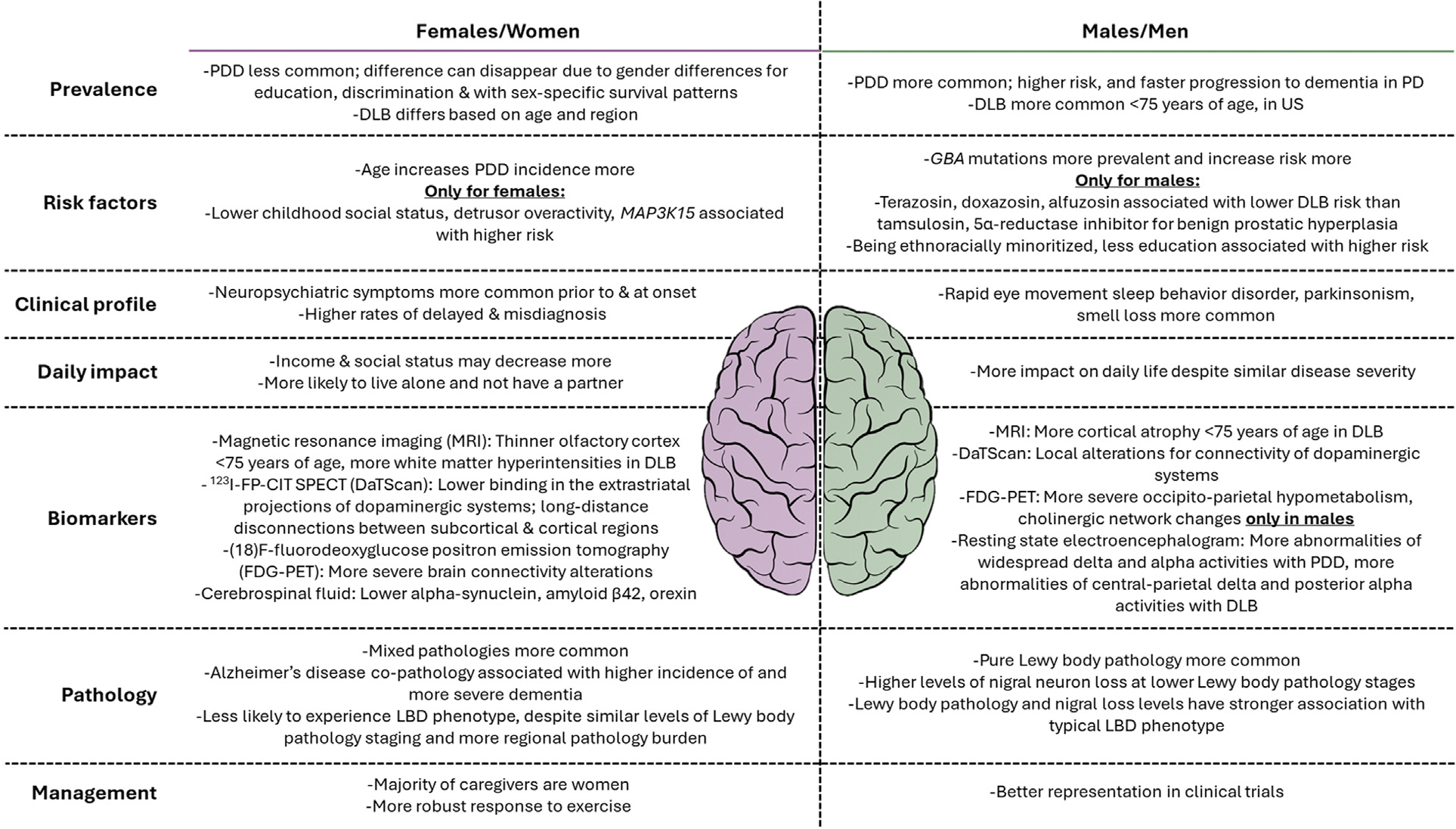
Overview of sex and gender differences reported for Lewy body dementia. DLB: dementia with Lewy bodies, LBD: Lewy body dementia, PD: Parkinson’s disease, PDD: Parkinson’s disease dementia.

So far, the literature in LBD supports sex differences from risk factors and biomarkers to clinical profile, treatment, daily impact, and pathology with differences across regions and age groups (Fig. 1). Majority of the studies have assessed sex, and not gender, with misuse of terminology at times. Although the aim was to also note findings for non-binary categories, no LBD studies focusing on intersex or non-binary genders were identified. The limited research focus on women continues to put them at risk for misdiagnosis and mismanagement. Understanding the sex and gender differences and identifying underlying reasons for these differences are needed for better diagnostic and therapeutic efforts for all.

## Figures and Tables

**Table 1:** Clinical profile for Parkinson’s disease dementia (PDD) [36] and dementia with Lewy bodies (DLB) [37].

	PDD	DLB

**Essential features for clinical diagnosis**	-Diagnosis of Parkinson’s disease -A progressive dementia syndrome that interferes with normal social or occupational functions or with usual daily activities	
**Cognitive features**	-Attention/working memory deficits -Cognitive fluctuations -Executive dysfunction -Visuospatial dysfunction -Memory decline -Language deficits -Processing speed deficits	
**Behavioral features**	-Visual hallucinations -Hallucinations in other modalities -Delusions -Rapid eye movement sleep behavior disorder -Excessive daytime sleepiness -Hypersomnia -Depression -Anxiety -Apathy -Personality changes	
**Motor features**	-Bradykinesia -Rest tremor -Rigidity -Postural instability and repeated falls -Slower gait	
**Autonomic and other features**	-Constipation -Orthostatic hypotension -Urinary incontinence -Sexual dysfunction -Syncope or other transient episodes of unresponsiveness -Abnormal sweating -Hyposmia	

**Table 2:** Likelihood of a typical dementia with Lewy bodies clinical syndrome based on the level of underlying Lewy, Alzheimer’s disease pathologies and substantia nigra neuronal loss [37].

		Alzheimer’s disease neuropathologic change (NIA-AA)		
	
		None-low/Braak NFT stage 0-II	Intermediate/Braak NFT stage III-IV	High/Braak NFT stage V-VI

**Lewy-related pathology**	Diffuse/neocortical	High	High	Intermediate
	Transitional/limbic	High	Intermediate	Low
	Brainstem-predominant; Amygdala-predominant; Olfactory bulb only	Low	Low	Low
**Substantia nigra neuronal loss assessed as none, mild, moderate, or severe to classify people into those likely or not to have parkinsonism** [95]				

NIA-AA: National Institute on Aging-Alzheimer’s Association guidelines for the neuropathologic assessment of Alzheimer’s disease [96]; NFT: neurofibrillary tangle.

**Table 3:** Pharmacological and non-pharmacological management approaches [1,75].

**Pharmacological options**	Cognition: Acetylcholinesterase inhibitors (donepezil, rivastigmine, galantamine), memantine Motor: Carbidopa-levodopa with dose adjustment to avoid psychosis and behavioral impairment ***AVOID amantadine, catechol-O-methyltransferase (COMT) inhibitors, monoamine oxidase inhibitors, dopamine agonists with high selectivity for the D2 receptor, anticholinergics for motor. They can exacerbate cognitive and behavioral impairment*** Behavior: Pimavanserin, clozapine with close monitoring for hematopoietic effects, low doses of risperidone, olanzapine in less severe cases ***AVOID benzodiazepines, tricyclic antidepressants, haloperidol due to serious side effects*** Sleep: Melatonin, low dose of clonazepam Orthostatic hypotension: Midodrine, droxidopa, fludrocortisone Urinary dysfunction: *β*_3_ agonists (mirabegron) ***AVOID antimuscarinic medications (oxybutynin, trospium). They can exacerbate cognitive impairment***
**Non-pharmacological options**	Exercise Healthy diet Psychotherapy Creative art therapies Mindfulness and meditation Support groups
